# Targeting pericytes for neurovascular regeneration

**DOI:** 10.1186/s12964-019-0340-8

**Published:** 2019-03-20

**Authors:** Mohammad Hossein Geranmayeh, Reza Rahbarghazi, Mehdi Farhoudi

**Affiliations:** 1 Research Center for Pharmaceutical Nanotechnology, Biomedicine Institute, Tabriz University of Medical Sciences, Tabriz, Iran; 2 Neurosciences Research Center (NSRC), Imam Reza Medical Center, Tabriz University of Medical Sciences, Golgasht St., Azadi Ave, Tabriz, 5166614756 Iran; 3 Drug Applied Research Center, Tabriz University of Medical Sciences, Tabriz, Iran; 4 Department of Applied Cell Sciences, Faculty of Advanced Medical Sciences, Tabriz University of Medical Sciences, Tabriz, Iran

**Keywords:** Pericytes, Blood-brain barrier restoration, Angiogenesis potential

## Abstract

Pericytes, as a key cellular part of the blood-brain barrier, play an important role in the maintenance of brain neurovascular unit. These cells participate in brain homeostasis by regulating vascular development and integrity mainly through secreting various factors. Pericytes per se show different restorative properties after blood-brain barrier injury. Upon the occurrence of brain acute and chronic diseases, pericytes provoke immune cells to regulate neuro-inflammatory conditions. Loss of pericytes in distinct neurologic disorders intensifies blood-brain barrier permeability and leads to vascular dementia. The therapeutic potential of pericytes is originated from the unique morphological shape, location, and their ability in providing vast paracrine and juxtacrine interactions. A subset of pericytes possesses multipotentiality and exhibit trans-differentiation capacity in the context of damaged tissue. This review article aimed to highlight the critical role of pericytes in restoration of the blood-brain barrier after injury by focusing on the dynamics of pericytes and cross-talk with other cell types.

## Pericytes role in structuring BBB and supporting during acute insults

**Fig. 1:**
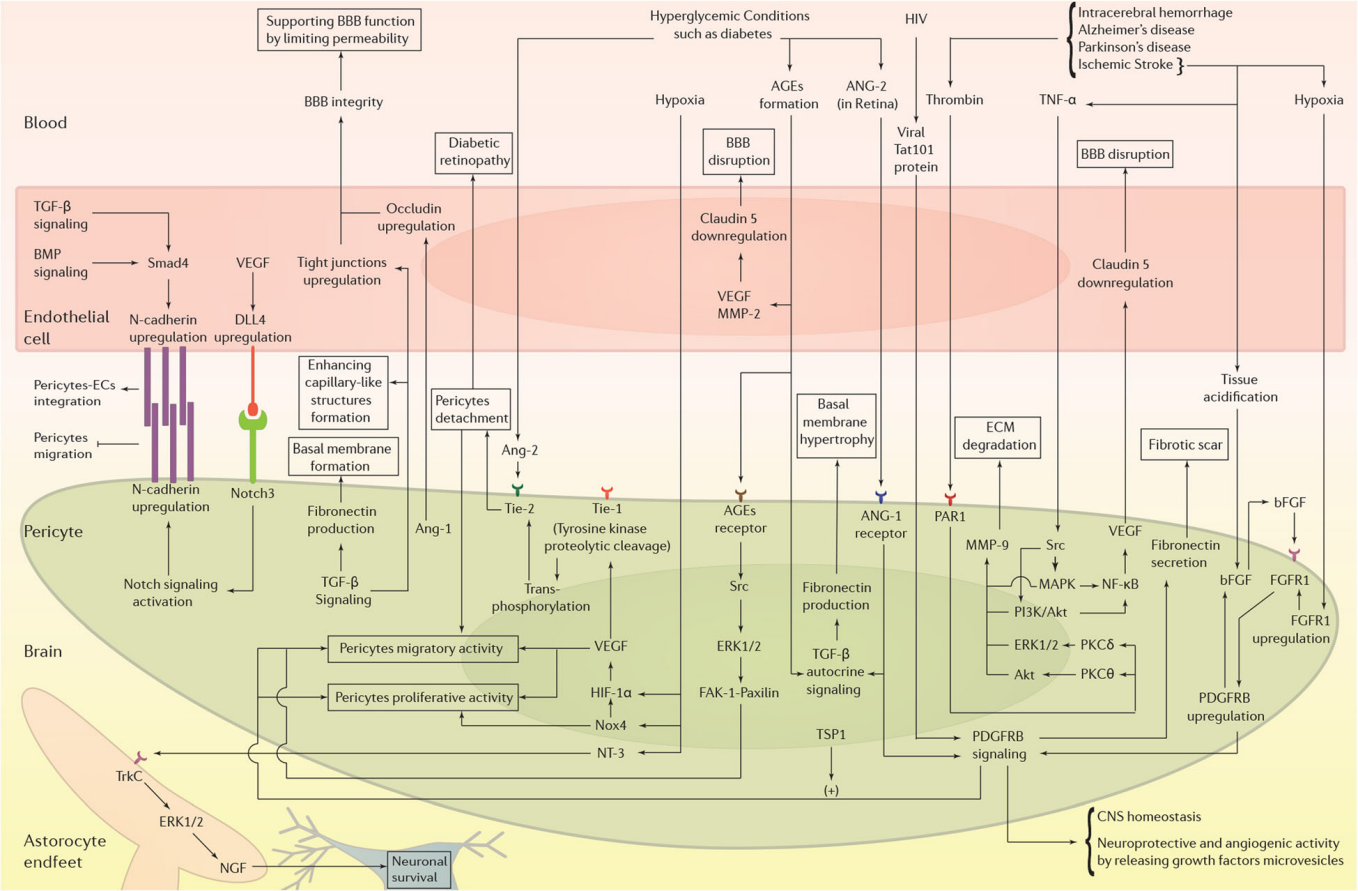
Pericytes contribute in homeostasis of the BBB through different mechanisms. TGF-β signaling inside pericytes supports the BBB integrity by enhancing fibronectin production, basal membrane formation and stimulating tight junctions expression. TGF-β signaling also participates in capillary-like structures formation. Along with TGF-β signaling, pericytes derived Ang-1 enhances occludin up-regulation inside ECs which stabilize BBB integrity. Mutually, ECs support adjacent pericytes by improving pericyte-EC integration by up-regulating N-cadherin and the prevention of pericytes migration. Two mechanisms have been suggested for ECs supporting role. First, TGF-β and BMP signaling pathways play enhancing role on N-cadherin up-regulation inside ECs through Smad4. The second mechanism is related to the stimulatory effect of VEGF in the expression of DLL4 inside ECs and attaching to receptor Notch3 on pericytes surface, triggering Notch signaling, N-cadherin up-regulation inside pericytes. Various mechanisms have been identified for the induction of pericytes proliferation and migration. During hyperglycemia or hypoxia, an elevated Ang-2 level activates cognate receptor Tie-2 which induces pericytes migratory activity by detaching cells from the ECM. The inductive mechanism for Ang-2 elevation in hypoxia occurs via pericytes HIF-1α and subsequent VEGF signaling. Also, the promotion of HIF-1α, VEGF, and Nox4 signaling after hypoxia enhances pericytes proliferative activity. In response to hypoxia, pericytes support neuronal survival with astrocytes collaboration. After hypoxia, pericytes NT-3 releasing activates astrocytes TrkC receptors in which upregulates NGF through ERK1/2 signaling pathway. Pericytes plays a major role in diabetic pathology and other hyperglycemic conditions. In these circumstances, pericytes respond to accumulated AGEs through various mechanisms. ANG-2-related activation of ANG type 1 receptor inside retina and AGEs stimulated TGF-β autocrine signaling inside pericytes, leading to basal membrane hypertrophy through increased production of fibronectin. The postulated mechanism for diabetic retinopathy via AGEs receptors occurs by activating downstream Src-Erk1/2-FAK-1-Paxillin signaling pathway which leads to diabetic retinopathy and pericytes migration. Also, HIV and ANG-2 cause pericytes migration and diabetic retinopathy through PDGF-BB autocrine signaling. Inside ECs claudin 5 down-regulation via VEGF and MMP-2 elevation leads to BBB disruption. Different types of CNS diseases such as ischemic stroke, intracerebral hemorrhage, Alzheimer’s disease, and Parkinson’s disease weaken BBB integrity through activating pericytes. By thrombin elevation in CNS diseases, PAR1 activation results in MMP-9 secretion and subsequent ECM degradation through downstream PKCδ-ERK1/2 and PKCθ-Akt signaling pathways. In addition, there are other mechanisms for MMP-9 release and ECM degradation after ischemic stroke related to increased TNF-α content and up-regulation MMP-9 inside pericytes through MAPK and PI3K/Akt signaling pathways. VEGF production inside pericytes happens and subsequent BBB disruption occurs through decreasing claudin 5 expression. In spite of these disruptive mechanisms of pericytes in response to ischemic stroke, pericytes behavior complexity stigmatizes its role by exerting CNS homeostasis, neuroprotective and angiogenic activity after ischemic stroke. The hypoxia-induced FGFR1 up-regulation and tissue acidification promotes bFGF autocrine signaling inside pericytes following ischemic stroke which intensifies PDGFRB up-regulation. PDGFRB signaling activation supports CNS homeostasis, neuroprotection, and angiogenesis through releasing growth factors microvesicles and generating fibrotic scar. TSP1 reinforces PDGFRB signaling aiming to pericytes proliferation and migration

A large number of people in developed societies are suffering from CNS problems burden them sensory, motor, and cognitive defects. BBB has the responsibility of maintaining homeostasis inside CNS by providing oxygen, nutrition, systemic regulatory factors, and removing metabolic wastes. Due to its complex and integrated structure, BBB delicately controls the crossing of chemicals on two sides of blood and brain. More importantly, BBB has a critical role in maintaining brain normal function in response to variations of multiple factors. Structurally, BBB is made up of different cell types, including ECs, pericytes, astrocytes, neurons, and microglia. The tight junctions between ECs strictly confine molecules transmission toward brain side (Fig. 1) [1–4].

On the blood side, BBB is paved by a united plane of ECs. The existence of tight junctions makes these cells to efficiently restrict molecules entrance toward the brain tissue. This characteristic in ECs is related to the exclusive expression of adhesion molecules such as claudins-3, − 5, and − 12. As a matter of fact, a main function of the BBB is to provide EC-EC integrity and various physical interactions between ECs with other resident cell types in neurovascular unit [2, 5, 6]. In addition to ECs, pericytes attach to the basal membrane matrix on both sides [7, 8]. Astrocytes connect to the basal membrane matrix through cellular endfeet adjacent to pericytes and ECs [9]. However, the supportive role of astrocytes highlights their prominent role in the establishment of long-lasting tight junctions by secreting trophic factors [10–12]. Additionally, astrocytes exert a protective role via releasing antioxidant-associated molecules for soothing neurodegenerative circumstances [10]. Microglia cells, as the main immune cell source inside CNS, are activated in response to brain injury [13, 14]. Based on data from previous experiments, microglia show two distinct phenotypes termed pro-inflammatory (M1) and anti-inflammatory (M2) microglia. During the occurrence of neuro-inflammatory status, the M1 phenotype augments immune response against insulting agent [13]. Increase in the M1/M2 ratio could promote a harmful effect on BBB integrity via modulating the dynamics of pro-inflammatory cytokines such as TNF-α (Fig. 1) [14]. In contrast, the activation of microglia M2 phenotype alleviates inflammatory response and promotes BBB regeneration by secreting protective factors [13]. Noteworthy, neurovascular adjacent neurons provide a supportive role in maintaining the physiological function of the BBB. In support of this claim, it was previously elucidated that neurons in the co-culture system enhanced tight junctions stability in the ECs by proper regulation of occludin [15, 16]. Accordingly, discovering reciprocal communication between the cells inside the BBB can be a promising approach for designing effective therapies for brain diseases. Regarding above descriptions, pericytes owe critical role in the context of BBB homeostasis and the identification of various aspects of pericytes behavior can be a remarkable target for manipulating healing procedure and acceleration of brain reconstitution.

The highest pericytes density exists in the CNS; thereby the brain has great potency for developing BBB integrity through the promotion of tight junction proteins between ECs [17–20]. Generally, pericytes are identified by the expression of NG2 chondroitin sulfate, PDGFRβ, CD146, and vimentin [17, 21–23]. Gap junction protein connexin-30 is exclusively seen in brain pericytes [24]. In muscular tissues, two subtypes of pericytes were identified from the aspect of absence or presence of Nestin. It was elucidated that type II pericytes are able to express Nestin while type I pericytes are Nestin-free. Considering different morphologies between the type I and type II pericytes, type I pericytes have potential to trans-differentiate into adipogenic and fibrogenic lineages. In contrast, type II pericytes possess myogenic, neurogenic, and angiogenic potential [23, 25, 26]. Even after neuronal differentiation, type II pericytes preserve the capacity of expressing Nestin, NG2, and CD146 [26]. Type II pericytes differentiation into neuronal and ECs lineages demonstrates an inherent heterogeneity between pericyte subtypes which highlights the presence of a diverse mechanism of response for even one type cell. This issue specifically supports brain options to respond to extensive disease-dependent conditions from demanding vascularization after brain injuries to limit vascularization in tumors [25, 27]. Based on another classification system, pericytes are divided into two subtypes based on desmin content. In type A pericytes, they become desmin negative and α-SMA positive cells five days after injury while type B pericytes express both factors. It has been shown type A pericytes secret a large amount of ECM on basal lamina compared to type B pericytes. Noteworthy, a high rate of migration and proliferation are seen after spinal cord injury by type A pericytes but not type B [28]. Collectively, it remains to determine the exact relationships and cross-talk between two types of pericytes under various conditions. PDGFRβ, a tyrosine kinase receptor, is up-regulated during embryogenesis in different cellular lineages along with mural cells [29]. A crucial role of PDGFRβ signaling has been determined in pericytes. Previously, it was shown that PDGFRβ signaling pathway promoted a cell behavior. For instance, upon the activation of PDGFRβ signaling, pericytes are able to proliferate, migrate to the injured sites. Additionally; numerous growth factors induce pericytes paracrine activity by using the PDGFRβ signaling axis.

A higher density of angiocrine factors and neurocine mediators are released via microvesicles in a paracrine manner such as brain-derived neurotrophic factor and β-nerve growth factor [17, 30]. One of the benefits achieved by microvesicles is these nano-scale sized particles can travel to remote sites in order to improve well-organized neuroprotection and angiogenesis. These effects are prominently blunted in the presence of a tyrosine kinase receptor inhibitor such as Sunitinib, confirming the key role of PDGFRβ in regenerative potential of pericytes [30]. Astrocytes endfeet are required for gliovascular formation and normal function of the BBB. Pericytes are found to facilitate astrocytic endfoot attachment to BBB. In support of this statement, the normal function of aquaporin 4, an astrocytic endfeet marker, is abolished in pericyte-deficient mouse mutant [31]. In addition, astrocytes could modulate angiogenesis potential of pericytes [32]. The migration and juxtaposition pericytes to ECs is controlled by its interaction with astrocytes which maintains normal vascular maintenance and homeostasis [33].

Pericytes can regulate BBB resident cells function through secreting various proteins in BBB (Table 1). The molecular interaction of pericytes with adjacent ECs per se regulates the homeostasis and integrity of the BBB [22]. Pericyte-EC physical contact increases trans-endothelial electrical resistance up to four times [17]. Results from experiments on pericyte-deficient mouse mutant showed a direct relationship between the BBB permeability and the number of pericytes [31]. Armulik and colleagues reported an increased BBB leakage in pericyte-deficient transgenic mice due to the lack of stabilizing effect of pericytes on the ECs [33]. The absence of pericytes, in turn, promotes the up-regulation of Ang-2, plasmalemma vesicle associated protein, and leukocyte adhesion molecules occur which elevates BBB permeability [17]. Noteworthy, these effects have not been observed in fibroblast barrier models, indicating tissue-specific interactions between pericytes and ECs [33]. By expressing a large amount of α-SMA, pericytes are able to control blood flow inside the brain microvascular system [18, 21]. It has been shown vessels expressing a high level of α-SMA are more permeable probably due to their potential ability to release a large content of VEGF, MMP-2, MMP-9, and bFGF [20, 22].

**Table 1:** Pericytes secretome on the regulation of astrocytes, neurons, microglia, and endothelial cells

Protein	Target cells	Signaling pathway inside pericytes	Signaling pathway inside target cell	Outcome	Ref.
NT-3 and NGF	Neurons and astrocytes	PDGFRβ-Akt		Neuroprotection after ischemic stroke	[112]
Pericyte-derived soluble factors	Hypothalamic neurons	ND^a^	Enhancing insulin-induced Akt phosphorylation	Raising insulin sensitivity	[113]
NT-3	Astrocytes	ND	TrkC-Erk1/2 signaling pathway	NGF production and subsequent neuronal survival during hypoxia	[35]
Agrin (possibly)	Astrocytes	ND	Regulating aquaporin-4 anchoring to perivascular endfeet	Enhancing fluid transport	[31, 114]
Bone morphogenetic protein 4	Oligodendrocyte precursor cells	ND	ND	Differentiation into astrocytes	[115]
Interleukin-6	Microglia	TNF-α activated Janus family of tyrosine kinase -STAT3 and inhibitor kappa B–NFκB pathways	iNOS mRNA up-regulation	Microglial proinflammatory activity	[116]
TGF-β1	Immature vessels ECs	ND	ND	Endothelial CD-146 down-regulation during maturation	[117]
Angiopoietin-1	ECs	ND	Tie-2 tyrosine phosphorylation	Occludin up-regulation	[118]
Monocyte chemoattractant protein-1	ECs	ND	ND	Enhancing HIV-1 transcytosis	[119]
TGF-β, VEGF, and MMP-2	ECs	AGE induced NF-κB signaling	Claudin-5 downregulation	BBB disruption	[51, 60]
Serpin protease nexin-1	ECs		Anti-thrombin	Inhibiting fibrinolysis	[120]
IL-8	Neutrophils	Stimulation by LPS, TNF-α, and IL-1β		Neutrophils chemo-attraction	[48]

^a^*ND* none determined

The primary response against oxygen and glucose deprivation is initiated via engaging STAT3, contributing to the control of metabolism and angiogenesis via bound regulated genes [34]. In hypoxic condition, pericytes showed a higher expression of NT-3 in which up-regulates astrocytes-derived NGF by the activation of the TrkC-Erk1/2 axis. This signaling pathway protects neurons during ischemic changes (Fig. 1) [35]. Under the hypoxic conditions, the entire pattern of microRNAs expression is altered inside pericytes to efficiently adapt the hypoxia. For example, the expression of pro-apoptotic miR-24 is reduced which is coincided with up-regulation of anti-apoptotic miR-345-5p. The transcription of miR-145 and miR-140 promotes cellular differentiation. However, these events happen by contradictory effects of TGF-β on miR-145 and miR-140. MiR-376b-5b induces pericytes differentiation and neovascularization under hypoxic conditions. These data support a notion that hypoxia induces pericytes stemness through distinct signaling pathways. However, it was shown miR-149-5p level declines pericytes migration rate and BBB permeability through elevating N-cadherin expression [36]. HIF-1α stimulates the level of VEGF, resulting in an enhanced pericytes proliferation and migration and subsequent angiogenesis. VEGF also elevates the content of miR-150 and miR-126 [37]. Along with these adaptations, hypoxic stress increases Nox4 and this enzyme promotes pericytes proliferation and ROS production [38, 39]. In severe hypoxia, the induction of caspase-3 hurts immature cortical pericytes [40].

Considering a supportive role of pericytes in BBB integrity after stroke, this capacity is impaired by genetic defects in *FOXF2* expression which reduces pericytes differentiation [41]. Pericytes have the capacity of identifying responses after stroke. The amount of PDGFRβ^+^ cells is elevated in the blood of a patient with acute stroke [42]. In response to an ischemic condition, bFGF expression increases inside pericytes which leads to autocrine and paracrine up-regulation of pericytes PDGFR-β. The absence of PDGFR-β signaling could promote brain hemorrhage because of microvessels aneurysm. In contrast, PDGF-BB/PDGFRβ signaling protects BBB and provides the regeneration of post-ischemic infarcted regions through enhancing pericytes recruitment. The mechanism is governed by bFGF which can be blocked by FGFR and PDGFR inhibition [43]. The BBB impairment after stroke is related to pericyte-derived VEGF. It has been shown using sodium cyanide, as a VEGF inducer inside pericytes, increases BBB permeability by down-regulating claudin-5 expression. In such a condition, tyrosine kinase Src is activated and initiated MAPK or PI3K/Akt signaling pathways leading to NF-κB activation mediating VEGF expression. Actually, increased permeability of BBB after sodium cyanide-induced VEGF expression has been abolished by VEGF blocking antibody [44]. Upon the occurrence of stroke, pericytes number is increased and the secretion of ECM is enhanced, resulting in the formation of discrete fibrotic scar different than glial scar [45]. It is postulated that PDGFRβ signaling triggers fibrosis through enhancing pericytes proliferation, differentiation into fibroblast-like cells, and secretion of ECM substrates such as fibronectin and collagen type I, subsequently (Fig. 1). Excessive or defective fibrosis can impair regeneration, so it should be regulated subtly after injury [46].

Thrombin forced pericytes to secrete MMP-9. This effect occurs through activation of thrombin PAR1 which follows by separate activation of PKCθ-Akt and PKCδ-ERK1/2 pathways and leads to MMP-9 production. As a result, the control of the BBB leakage in pro-inflammatory conditions could be achieved by limiting the capacity of pericytes to release MMP-9 in response to raised levels of thrombin following ischemic stroke, intracerebral hemorrhage, Alzheimer’s disease, and Parkinson’s disease (Fig. 1) [8, 47]. Similar to thrombin, TNF-α can induce the production of MMP-9 inside pericytes [48, 49]. The critical role of pericytes in restricting neuro-inflammation and immune response has been shown in multiple sclerosis. During this circumstance, the down-expression of ALCAM inside vascular cells contributed to an enhanced movement of leukocytes across ECs [17]. In the occurrence of different pathologies, the mutual interaction of pericytes and astrocytes increase the expression of tight junction proteins. Astrocytes have an inevitable role in regulating BBB integrity in acute phases of inflammation while the critical role of pericytes is shown in the late phases of inflammation [32]. The over-activation of angiogenic response, as seen in the hypoxic condition, has a detrimental effect on the normal function of BBB by engaging the VEGF/VEGFR-2 signaling pathway [19]. In contrast, the activation of PDGF-BB/PDGFRβ signaling in pericytes protects rodents CNS after stroke. In Parkinson’s disease, the secretion of bFGF could improve BBB function [30]. bFGF enhances the expression of PDGFRβ inside pericytes which has regulatory feedback and triggers interplay between endogenous growth factors for serving CNS homeostasis (Fig. 1) [43].

## Reactive and inflammatory role of pericytes during chronic diseases

Loss of pericytes in neurologic diseases increases BBB permeability. For instance, during diabetes mellitus, BBB integrity mitigates and results in vascular dementia. Due to pericytes restorative role, they can ameliorate the detrimental effects of diabetes mellitus inside the brain [7, 50]. Some authorities showed the critical role of PDGFRβ signaling in brain pathologies. This signaling pathway is committed to preserving the normal amount of pericytes around vessels. In the condition with a decrease of PDGFRβ signaling activity, a simultaneous reduction in pericytes numbers is observable with the emergence of vasodilation in the neurovascular unit [50–52]. α-SMA positive pericytes death happens in the absence of cathepsin D as described in congenital neuronal ceroid disease through producing mitochondrial ROS and lysosomal dysfunction [50]. Exposing pericytes to bacterial lipopolysaccharides induces their immunoreactivity and detachment from the basal lamina. Along with these changes, pericytes show morphological changes after microglial activation. Toll-like receptor-4 is up-regulated after pericytes exposure to lipopolysaccharides and suggested to be responsible for above-mentioned changes [53]. Attachment to underlying basal lamina controls the dynamics of pericytes. Based on data from experiments, cell detachment from basal lamina, as seen during ischemic stroke and traumatic injuries, leads to pericytes loss [45, 53]. Connexin-43, as a major gap junction protein between pericytes, has a distinct role in the pathophysiology of some pericyte-related diseases. Different pathologies have been correlated with the function of connexin-43 during various diseases. Notably, the human immunodeficiency virus enhances its spreading inside the brain by elevating the up-regulation of connexin-43 [54]. Conversely, it has been suggested connexin-43 down-regulation can be a role player in diabetic retinopathy. The postulated mechanism is attributed to the dense co-localization of ECs claudin-5 and pericytes connexin-43 [55].

TGF-β has multiple roles in directing pericytes to have normal function. This factor regulates pericytes morphological feature, specific marker expression, and differentiation capacity [18]. Fibronectin production, a basal membrane component, is increased by TGF-β1 autocrine signaling and maintains BBB proper function (Fig. 1) [20, 51]. In the co-culture system of pericytes and ECs, the formation of capillary-like structures was enhanced via TGF-β1 secretion [56]. Furthermore, BBB damage and neurotoxicity were observed following cyclosporine administration. This event is attributed to the inhibitory effect of cyclosporine on pericytes TGF-β1 secretion [57]. The importance of TGF-β1 signaling inside pericytes has been highlighted in loss of Smad4, as a pivotal mediator of TGF-β1, causing the advent of neonatal intracranial hemorrhage [58].

Previous works provided evidence that pericytes autocrine capacity could be distorted during metabolic diseases such as diabetes. In diabetic and hyperglycemic conditions, the formation of AGEs contributes to the disruption of BBB integrity by pointing ECs in which claudin-5 down-expression occurs by up-regulation of MMP-2 and VEGF. Here in, AGEs also target pericytes by enhancing their TGF-β autocrine signaling resulting in fibronectin production and basement membrane hypertrophy and raising their migratory activity (Fig. 1) [51]. At the time of infection with Japanese encephalitis virus or emergence of inflammatory conditions, BBB leakage is intensified by infected pericytes which have negative effects on ECs integrity. The virus has an affinity to toll-like receptor 7 and activates downstream effector TAK1, leading to the activation of NF-κB. The activation of effector TAK1 increases the phosphorylation of cytosolic phospholipase A2 via the ERK signaling pathway and prostaglandin E2 content. Autocrine activation of pericytes prostaglandin E_2_ receptor-2 is enhanced by the promotion of NF-κB through cAMP-dependent protein kinase A. These conditions increase ECs interleukin 6 content and the expression of chemokine ligand 5. Both of the molecules have the ability to induce BBB permeability and increase trans-endothelial leukocyte migration rate, respectively [59].

The pericyte-EC close physical contact is essential for controlling BBB integrity and function. Actually, it has been suggested juxtacrine contacts between both cell types are inevitable for normal development of the BBB [21]. In addition to angiogenic activity, pericytes have an anti-angiogenic effect on endothelial lineage by decreasing ECs proliferation rate [21]. LPS or cytokines were found to make pericytes recall chemo-attract neutrophils by releasing IL-8. Furthermore, MMPs secretion supports IL-8 activity via attenuating neutrophils adhesion to pericytes. This issue provides a condition for neutrophils transmigration across the BBB [48].

Integrity of the BBB could be reduced by pericytes upon the secretion of VEGF and MMPs [22, 48, 60]. By contrast, pericytes secrete Ang-1 and TGF-β which has an important role in supporting EC-EC tight junctions that maintains the integrity of the BBB (Fig. 1) [18, 53, 60]. Ang-1 is assumed to decrease the permeability of BBB through up-regulating endothelial cells occludin [20, 33]. The release of angiotensin-converting enzyme 2 and ECM proteins such as fibronectin and collagen type IV are also regulated by TGF-β [17, 51]. As mentioned before, Smads downstream signaling effector, TGF-β, plays a key role in crosstalk between pericytes and ECs. Study on Samd4-deficient ECs revealed a decline in Notch receptors cellular distribution and N-cadherin expression. As a matter of fact, the connection between pericytes and ECs faints which leads to intracerebral hemorrhage occurrence in mouse neonates. Under physiological conditions, TGF-β and BMP signaling collaborate with Notch pathway boosting N-cadherin up-regulation and subsequent integration of pericytes with ECs [58]. Actually, a pivotal role of Notch signaling emerges physical attachment of the pericytes-EC complex. EC-derived Notch ligand DLL4 activates Notch3 receptors on brain α-SMA positive pericytes and reduces pericyte migration and angiogenic function. Commensurate with this statement, genetic mutation of DLL4 promotes is the main reason for cerebral cavernous malformation disease [61–64].

The migration of pericytes is deeply influenced by their interaction with ECs. Pericytes migration is preceded by the release of proteases and degradation of ECM [65]. During neovascularization, pericytes release PDGF to manage the in vivo tube formation by recalling ECs [66]. Migration is prompt through the reciprocal collaboration between FAK-1 and paxillin inside the pericytes. Upon the occurrence of a diabetic condition, the loss of retinal pericytes is regarded as the reason for vascular retinopathy. As seen in diabetes mellitus, the surplus amounts of AGEs activate the corresponding receptor, promoting Src-ERK1/2-FAK-1-paxillin signaling pathway which leads to pericytes migration and an abrogated vascular integrity (Fig. 1). Pericytes migration rate can be reduced by the presence of AGEs-induced through the application, ERK1/2 phosphorylation inhibiting molecule [67]. Another proposed mechanism for pericytes migration in diabetic retinopathy is related to Ang-2 and its receptor Tie-2 activation via hyperglycemia-induced elevation of the main product of AGEs named methylglyoxal (Fig. 1) [68–70]. Furthermore, VEGF-induced Tie-2 activation through Tie-1 tyrosine kinase proteolysis following by Tie-2 trans-phosphorylation can be regarded as another mechanism for pericytes migratory behavior [71, 72]. With the progress of chronic inflammation, the production of interferon-gamma mainly diminishes pericytes mobilization through preventing PDGF-BB-mediated PDGFR-β signaling [73].

Another factor termed TSP1 has paradoxical effects on ECs and pericytes. TSP1 prevents ECs migration while enhances pericytes mobilization. Actually, pericyte responsiveness to pro-migratory stimulation of PDGF is depended on the presence of TSP1. This effect was suppressed in TSP1 deficient pericytes (Fig. 1) [74]. The higher concentration of angiotensin-II in the retinopathy compared to the plasma can enhance retinal pericytes migration through PDGF-BB and TGF-β pathways. The pro-migratory effect of angiotensin-II can be inhibited by blocking its type 1 receptor antagonists but not type 2. So, type 1 antagonists such as losartan or angiotensin-converting enzyme inhibitors can be useful in diabetic retinopathy. In spite of the inevitable role of MMPs as a basic factor for cell migration, the suppression of MMPs activity did not affect the migratory effect of angiotensin II. Haptotaxis is suggestible as a migration mechanism which is governed by cell adhesion to collagen substrate [75, 76]. In the case of hypoxia, pericytes-derived angiopoietin-1 enhances ECs migration and tubulogenesis [77]. The critical role of PDGF-BB has been found in brain pericytes. It has been shown that brain microvessel pericytes loss occurs during human immunodeficiency viruses infection. Viral Tat101 protein is responsible for enhancing pericytes migratory activity through PDGF-BB up-regulation and subsequent autocrine signaling of PDGFRβ signaling (Fig. 1) [78]. PDGF-BB signaling on the migration of pericytes can be inhibited by activating Roundabout 1 receptor via Slit2 which is present in axonal growth cones [79].

The balance of neural activity and cerebral capillaries blood flow guarantees proper function of the brain which termed neurovascular coupling. Pericytes motile identity introduces them as a prominent regulator of the BBB. During repeated seizures, neurovascular insufficiency occurs because of pericytes damage. Probably, mitochondrial injury underlies as a foremost cause of pericytes dysfunction following recurrent seizures which regulates intracellular Ca^2+^ needed for pericytes motility and contraction [80]. Here in, BBB pericytes loss or their ectopic coverage is seen in status epilepticus [81, 82]. Therefore, pericytes hypertrophy and detachment is observable in epileptic human cortical specimens. As a part of complexity, in seizure disorders, pericytosis accompanies by co-localization of microglial aggregation in perivascular areas which debilitates BBB function. Considering the stimulatory role of pro-inflammatory cytokines in pericytosis, microglial immune response to inflammatory cytokines might mediate pericytes redistribution and pericytosis progress in epileptic pathologies, however, underlying mechanisms need to be deciphered [83]. Microglial pro-inflammatory M1 phenotype shifting to anti-inflammatory M2 phenotype [84] is a suggestable approach to understand microglia contribution in pericytosis following epilepsy.

PDGFRβ/NG2 cells redistribution and higher immunoreactivity of PDGFRβ are observable in epileptic specimens. PDGFRβ signaling could be attributed as an enhancer of pericytes migratory activity [73, 85, 86]. However, utilizing PDGF-BB prevents loss of pericytes and vascular smooth muscle cells (another type of mural cells) in seizure induced-brain. Hence, PDGFRβ signaling mitigates BBB dysfunction and epileptiform electroencephalogram activity. The amount of murals cells redistribution is related to vascular diameter in resting condition [87]. This issue not only highlights PDGFRβ signaling as a response against seizure disorders for maintaining CNS homeostasis but also stigmatizes pericytes neuro-protective role via releasing microvesicles containing growth factors [30]. Along with pericytes direct participation in epileptic pathology, indirectly, pericytes loss raises BBB permeability permitting serum proteins influx into brain parenchyma which enters inside astrocytes through TGFβ receptors. In response, Kir4.1 (potassium inward rectifier) and glutamate transporters down-regulation occur which increases neuronal excitability promoting epileptogenesis [88].

## Pericytes restorative and stem cell-like behavior in BBB recovery

BBB resident pericytes show similarities with MSCs from the aspect of cell surface proteins expression and cellular behavior. Based on data from an experiment done by Tian et al., they showed treatment of MSCs, instead of pericytes in in vitro model of the BBB, with TGF-β or collagen type I elevated CD146 expression [18]. The role of pericytes in the promotion of angiogenesis and neovascularization are proved in the recovery of damaged BBB [48, 89, 90]. TNF-α is found to increase pericytes migration during inflammatory conditions by ECM degradation. TNF-α exerts this effect through up-regulating MMP-9 inside pericytes [49]. Therefore, controlling interactions of TNF-α with MMP-9 during pro-inflammatory conditions can be an important approach for preventing pericytes loss and subsequent BBB dysfunction after neurodegenerative states. The potential role of pericytes on the differentiation of oligodendrocyte progenitor cells by Lama2 confirms pericytes as a tool for improving re-myelination in diseases such as multiple sclerosis [91]. Pericytes have shown to be confronting atherosclerosis expansion by regulating shear stress responses of ECs. In response to shear stress, ECs up-regulate ADAMTS-1, contributing to ECM turnover and atherosclerosis development. Interestingly, pericyte-derived ADAMTS-1, in turn, up-regulates tissue inhibitors of matrix metalloproteinases which inhibits ADAMTS-1 expression inside ECs. This data introduces pericytes as an accessible candidate for alleviating shear stress during atherosclerosis by supporting vessels stability [92]. Moreover, enhancive effects of pericytes on vascularization were shown in the co-culturing system by application of fibroblasts with endothelial progenitor cells and MSCs on acellular human dermis [93].

Principally, two different aspects of pericyte-derived angiogenesis must be considered in the context of CNS. In brain tumors, the pro-angiogenic capacity of pericytes should be inhibited while during brain injury pro-angiogenic capacity of these cells is better to promote. The angiogenesis process is tightly controlled by both pro-angiogenic and anti-angiogenic factors [94]. It has been touted that the pro-angiogenic potency of VEGF-B was exerted on pericytes by enhancing expression of IL-8, PAI-1, SDF-1, and macrophage migration inhibitory factor through various intracellular mechanisms. For instance, the expression of both CXCR-1 and CXCR-2 were induced after ECs exposure to IL-8. PAI-1 has the potential to blunt uncontrolled angiogenesis. SDF-1 recall bone marrow CXCR4^+^ cells to neovascularization zones, so this mechanism can contribute angiogenesis after brain ischemia [94].

The support of neurogenesis is another mechanism for pericytes toward regeneration. Pericytes might regulate neurogenesis indirectly through the synthesis of ECM proteins which take part in the presenting regulatory molecules to stem cell niche through tethering mechanism [95]. In ischemic conditions, pericytes precede neurogenesis by secreting factors including, C-X-C motif chemokine ligand 12, NGF, and NT-3 [96]. With the continuation of neurogenic response, the release of CORM-3 has shown to intensify neurogenesis through nitric oxide cross-talk between pericytes and NSCs following traumatic brain injury. The phosphorylation of NSCs nitric oxide signaling is governed by CORM-3 [97].

**Fig. 2:**
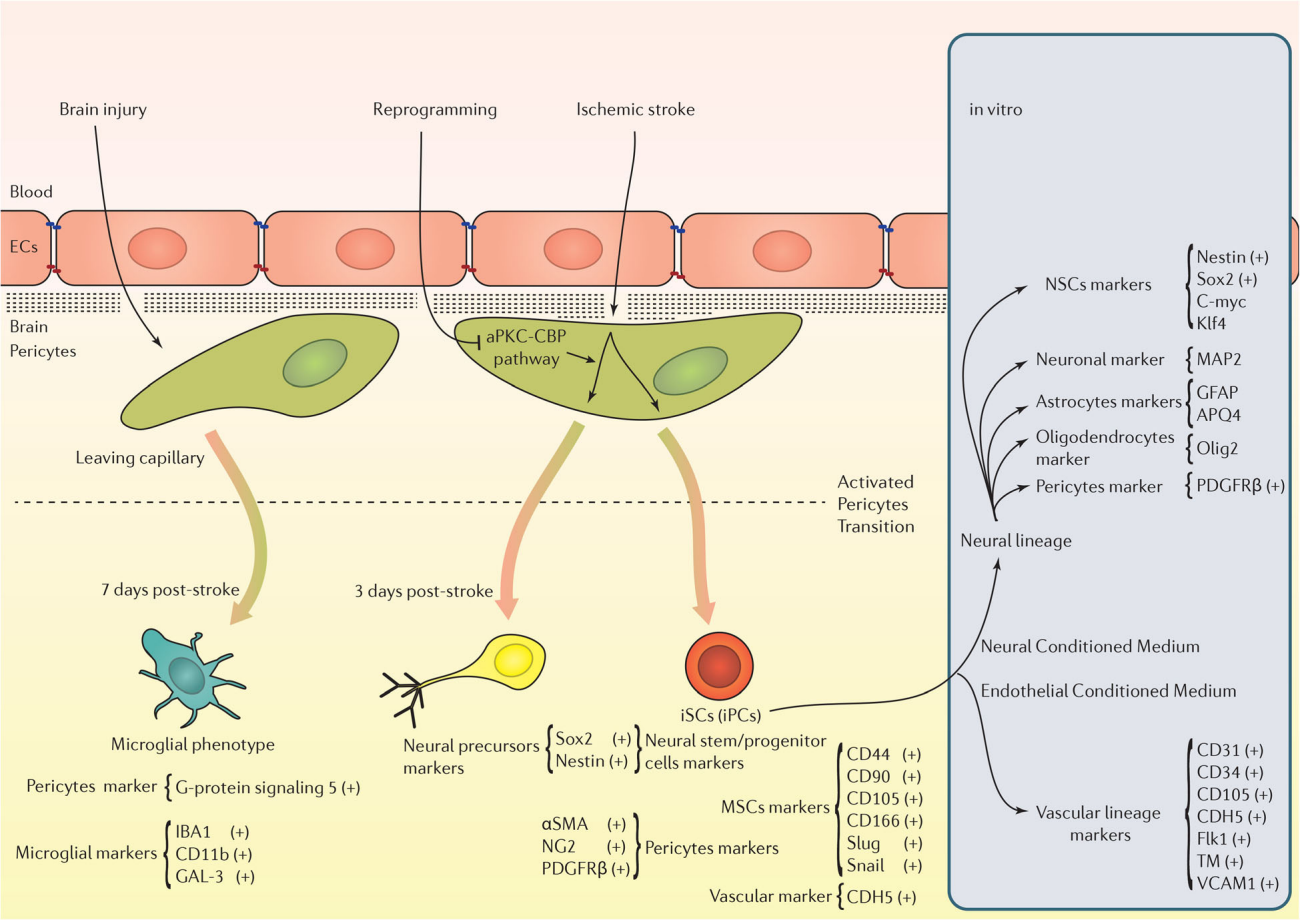
Under pathologic circumstances, pericytes present stemness or trans-differentiation potential. Brain injury induces pericytes to leave capillary and achieve microglial phenotype while pericyte marker expression retains. After ischemic stroke, the suppression of the pericytes aPKC-CBP signaling pathway contributes in reemergence of induced neural precursors markers 3 days post-stroke. However, iSCs generation in ischemic regions pericytes is plausible by co-expressing MSCs, Neural stem/progenitor cells, and vascular lineage markers along with pericytes markers. Utilization of neural- or endothelial-conditioned medium on iPCs culture in vitro leads to neural or vascular lineage generation

Upon the occurrence of brain pathologies, pericytes provoke immune cells to regulate neuro-inflammatory conditions. Even though, it was shown that pericytes are capable to represent microglial-like phenotype [30]. Notably, pericytes can acquire microglial phenotype in ischemic brain injuries after leaving capillary which has been demonstrated by tracking G-protein signaling 5 as a pericyte specific marker in microglial cells [98]. This feature represents pericytes as a potential source of microglial cells. Furthermore, microglia can be targeted for adopting neuroprotective phenotype of M2 microglia (Fig. 2). After a focal ischemic stroke, pericytes reveal reprogramming capacity for generating neural precursors through inhibiting aPKC-CBP signaling pathway. This mechanism is not beneficial in the course of the chronic stroke phase as aPKC-CBP signaling pathway activation; however, it is essential for neurovascular renovation and functional recovery (Fig. 2) [99]. Generally, the presence of iPCs (iSCs) is identified by the simultaneous expression of stem cells, vascular lineage, and pericytes markers in the human post-stroke brain. These iPCs originate from pericytes and reside adjacent to brain capillaries in infarcted regions. Interestingly, utilization of neural- or endothelial-conditioned medium directs iPCs into a new destination of neural or vascular lineage, respectively (Fig. 2) [100]. Furthermore, direct reprogramming as a targeted approach for differentiated cells conversion into a favorite type of cells has been reported to be successful in pericytes trans-differentiation into neurons. By exploiting synergistic activity of Ascl1 and Sox2 transcription factors, pericytes conversion into neurons is detectable by lineage-specific genetic transcriptome up-regulation. Interestingly, neuron-derived pericytes have the potential of bifurcation into glutamatergic or GABAergic lineages opting development of new strategies for cell replacement therapies by achieving suitable proportions of neuronal subtypes [101, 102]. Exclusively, this issue highlights the importance of ischemic regions pericytes capability in inducing neurogenesis or their autologous transplantation in stroke patients [103].

As pericytes have pluripotent individualities, so they have the capacity to trans-differentiate into other cell types such mesenchymal lineage [21, 22], neurons, astrocytes, and oligodendrocytes [22, 29, 104]. Type A pericytes, but not types B, participate in scar tissue formation after spinal cord injury. They exert this effect through proliferation and moving along with vascular sprouting into injured tissue. This issue introduces scar formation as a natural behavior of pericytes against CNS injury [28]. As mentioned before, there are behavioral similarities between MSCs and pericytes such as regenerative and anti-inflammatory characteristics which gives hope for therapeutic outcomes in the future [30]. Pericytes could respond and adopt changes induced by LPS through expressing CD11b and CD146. These properties indicate microglial phenotype acquisition by pericytes during inflammatory situations. One explanation would be that pericytes release pro-inflammatory cytokines after exposure to LPS [30]. Recently, pericytes lineage plasticity in the pathological setting has been investigated by a fate-mapping technique in vivo. The results showed pericytes do not differentiate into other cell types during experimental injury or aging in which challenged general believes about pericytes exploitation in vivo [29]. Comparing restorative effects of transplanted pericytes with non-regenerative behavior of endogenous pericytes [29] highlights some principal questions about the dual behavior of pericytes in vitro and in vivo conditions. It shows that some responsible factors inside the culture environment probably play a critical role in differentiation and regeneration of pericytes. This point necessitates designing of feasible approaches for potentiating endogenous pericytes targeting for cell replacement therapy. In spite of pericytes supportive role in BBB recovery, their application in tumors can be risky as it can help tumor growth by stimulating vasculogenesis [105, 106]. In this regard, eliminating glioma stem cells-derived pericytes inhibits tumor growth and vascularization of glioblastoma [107].

## Conclusion and future outlooks

Because of particular characteristics, pericytes show a prominent role in protecting BBB during diseases which can be highly important for maintaining brain function [53]. In addition to the stroke, precise studies need to be designed for exploiting diverse and restorative capacities of pericytes in the recovery of diseases such as antioxidant behavior in amyotrophic lateral sclerosis [108], anti-aging behavior in Alzheimer’s disease. Improved brain function occurs after MSCs-derived pericytes implantation in mice model of Alzheimer’s disease [109, 110] through enhancing brain microcirculation and pericytes phagocytic clearance of Aβ deposits [111]. Easy accessibility of pericytes for autologous transplantation highlights their capabilities for future regenerative studies [27]. This issue supports the notion that pericytes can be a choice tool for targeting CNS recovery following injuries and diseases.

## Abbreviations

ADAMTS-1: A disintegrin and metalloproteinase with thrombospondin motif 1
AGEs: Advanced glycation end products
Ang-1: Angiopoietin-1
Ang-2: Angiopoietin-2
aPKC-CBP: Atypical protein kinase C-mediated phosphorylation of CREB-binding protein
BBB: Blood-brain barrier
bFGF: Basic fibroblast growth factor
BMP: Bone morphogenetic protein
CNS: Central nervous system
CORM-3: carbon monoxide-releasing molecule-3
CXCR: CXC receptor
DLL4: Delta-like ligand 4
ECM: Extracellular matrix
ECs: Endothelial cells
ERK1/2: Extracellular signal-regulated kinase 1/2
FAK-1: Focal adhesion kinase-1
FGFR: Fibroblast growth factor receptor
HIF-1α: Hypoxia-inducible factor 1 alpha
HIV: Immunodeficiency virus
IL-8: Interleukin-8
iNOS: Inducible nitric oxide synthase
iPCs: Ischemic region pericytes
iSCs: Ischemia-induced multipotent stem cells
LPS: Lipopolysaccharide
MAPK: Mitogen-activated protein kinase
MMP-2: Matrix metalloproteinase-2
MMP-9: Matrix metalloproteinase-9
MSCs: Mesenchymal stem cells
NFκB: Nuclear factor kappa B
NGF: Nerve growth factor
Nox4: NADPH oxidase 4
NSCs: Neural stem cells
NT-3: Neurotrophin-3
PAI-1: Plasminogen activator inhibitor-1
PAR1: Protease-activated receptor-1
PDGF-BB: Platelet-derived growth factor-BB
PDGFRβ: Platelet-derived growth factor β
PI3K: Phosphoinositide 3-kinase
PKCδ: Protein kinase C delta
PKCθ: Protein kinase C theta
PSCs: Pluripotent stem cells
ROS: Reactive oxygen species
SDF-1: Stromal-cell-derived factor-1
STAT3: Signal transducer and activator of transcription 3
TAK1: Transforming growth factor β-activated kinase-1
TGF-β: Transforming growth factor beta
TNF-α: Tumor necrosis factor alpha
TSP1: Thrombospondin 1
VEGF: Vascular endothelial growth factor
α-SMA: alpha-smooth muscle actin
